# Forty-Five Years after the Discovery of the Hepatitis D Virus: Where Do We Stand?

**DOI:** 10.3390/v13040555

**Published:** 2021-03-26

**Authors:** Mario Rizzetto, Tommaso Stroffolini

**Affiliations:** 1 Department of Medical Sciences, University of Turin, 10126 Turin, Italy; 2 Department of Tropical and Infectious Diseases, Policlinico Umberto I, University of Rome, 00161 Rome, Italy; tommaso.stroffolini@hotmail.it

## 1. The Discovery of the Hepatitis D Virus

The discovery of the Australia Antigen in the mid-1960s led, in a few years, to the identification of the virus of Hepatitis B. Shortly after, the Hepatitis A virus was discovered in 1973, filling the gap in the knowledge of the etiological agents of infectious and serum hepatitis, the existence of which had been predicted by epidemiological studies of previous decades.

By the mid-1970s it became clear that neither the hepatitis A nor the hepatitis B viruses accounted for a distinct proportion of transfusion-transmitted hepatitis, unleashing a search for an elusive non-A non-B hepatitis virus that engaged the hepatological community till the identification of the hepatitis C Virus 15 years later.

While the discovery of the hepatitis A, B and C viruses was fostered by a large body of previous epidemiological indications, the report in the late 1970s of a new hepatitis virus, originally named delta, came out of the blue into the virologic scenario of the time, as unexpected and unexplained.

The origin was a diagnostic discrepancy. Sophisticated molecular diagnostics were not available in the 1970s. To determine viral activity in HBsAg-positive liver biopsies, we looked, in Torino, at liver immunohistochemistry for the core antigen of the HBV (HBcAg). HBsAg-positive liver biopsies were challenged with a reference fluoresceinated antiserum to the HBcAg (anti-HBc), which elicited granular nuclear fluorescence in nuclei containing the antigen.

However, the amount of the precious reference reagent was limited and we were concerned with sparing it as much as possible. In the mid-1970s we found that the HBcAg in liver fixed human complement (fresh HBsAg-negative serum) added over cryostat-cut biopsy sections [1]. In nuclei containing the HBcAg, adding fluoresceinated antibodies against complement (C) proteins produced staining similar to that of the HBcAg, presumably because the intrahepatic antigen was complexed with the homologous antibody.

We started to utilize this simple test as an expedient to spare the precious reference anti-HBc serum. HBsAg-positive biopsies were first screened with the C fixation test, and the valuable anti-HBc serum was then used only in biopsies that fixed C.

However, we found soon that some HBsAg-positive liver diseases fixed C but did not react with the reference anti-HBc serum. Reasoning that these biopsies possibly contained immunocomplexes of another antigen, we labeled with fluorescein the IgG from the serum of a HBsAg-positive patient displaying the unexplained C fixation phenomenon and challenged his own liver biopsy. The suspicion was confirmed; the newly prepared antiserum reacted with an unknown antigen in the liver nuclei yielding a fluorescent staining similar to the HBcAg. In electron microscopy, no particle or structured virus-like elements were detected in the biopsy, and in immune-electron microscopy, peroxidase-conjugated antisera against the new antigen deposited on clumps of amorphous nuclear material.

We named the new reactivity the HBsAg-associated delta antigen and reported it in 1977 as a probably unrecognized component of the HBV nucleocapsid [2].

Unfortunately, our enthusiasm turned soon into frustration; the report was met with skepticism and the general incredulity meant that delta would die out alongside several other strange HBV subtypes published at the time.

However,”audentes fortuna juvat” (Virgilius: fortune favors the brave).

One of us (MR) made an application to the National Institute of Health in Bethesda to further study delta reactivity and was fortunate; the US institution became interested and offered a stage in 1978.

With the expertise and advice of master virologists such as Dr. James Shih, Dr. John Gerin and Dr. Robert Purcell, it was easy and quick at the NIH to develop a radioimmunoassay for the delta antigen-antibody system in serum, using as substrate an antigen-positive human liver. With a simple diagnostic tool at hand, experimental transmission studies were started in chimpanzees [3]. An HBV naive animal was inoculated with the serum of an Italian HBsAg-positive patient who had delta in liver and antibody to it in blood. After a delay consistent with the incubation period of an acute HBV infection, the HBsAg became detectable in the blood of the animal, followed by the appearance of delta in the liver, which increased while the animal was experiencing severe hepatitis, and ultimately cleared, together with serum HBsAg, in a few weeks. This experiment showed that delta was transmissible, but transmission was associated with raising of the HBV, in apparent confirmation of its HBV origin.

However, when the same infectious inoculum was injected into a symptom-free chimpanzee who had been carrying the HBsAg for a long period, liver cell nuclei displaying large amounts of delta were seen as early as one week after inoculation and persisted for long in the liver. In parallel with the colonization by delta, the HBsAg rapidly diminished in blood, and severe hepatitis occurred in the previously healthy animal [3].

This pattern of infection ruled out that delta was the component of an HBV variant, suggesting instead that it was the expression of a pathogenic, defective nonautonomous viral agent that required biological help from the HBV.

The animal model led to the recovery of large amounts of the delta antigen in blood, rapidly driving the identification and characterization of the infectious agent by which the antigen was expressed. Pelleting of serum by ultracentrifugation, and disruption of the pellet with detergent, revealed that the delta antigen was the internal component of a 36 nm amorphous particle, which was coated by the HBsAg and contained a very small RNA representing the genome of a new virus [4].

In keeping with the standard nomenclature of hepatitis viruses (waiting for the HCV), at the beginning of the 1980s the delta was renamed the Hepatitis D Virus (HDV), though the denomination “hepatitis delta virus” is still widely used [5].

## 2. The Last Forty Years

Virologic progress has been astounding. HDV is now considered a unique liver pathogen with biological properties unknown in animals, and unlike conventional viruses [6]. Though the evolution of HDV was thought to lie with humans in coevolution with HBV, delta-like viruses have now been found throughout the biological tree in the absence of an hepadnavirus, from invertebrates to fish, birds and reptiles [7]. The exciting biological and virologic developments of HDV, and its evolutionary perspectives, are addressed and detailed in this issue of Viruses.

Unfortunately, many epidemiological gaps and unmet clinical needs remain in the appraisal and management of hepatitis D. Though HDV is a liver pathogen of worldwide importance, international health agencies have long ignored its infection and the World Health Organization has recognized only recently the issue of hepatitis D [8]. The approach and modalities to HDV testing have not been addressed and standardized at public health levels. The number of infected patients remains uncertain. In three different metanalyses, global prevalences have varied from 12 to 72 million people [9,10,11], reflecting disparate recruitment methodologies and lack of data of sufficient quality. Because of low awareness and inadequate screening, the rate of case ascertainment is low and the overall contribution of HDV to the burden of HBsAg liver diseases remains undefined.

Field therapy has not gone further than the Interferon (IFN) treatment introduced empirically in the late 1980s. Big Pharma was not interested, in the perception that control of HBV with vaccinal prophylaxis would ultimately lead to the eradication of HDV in the developed world, and because of lack of economic incentive in a disease prevailing in disadvantaged regions of the world with poor financial resources. The task of therapeutic research has been left to inventive research institutions and small enterprises with limited resources. However, awareness and diagnostic facilities have improved in recent years, and the prospect of new therapies has raised the need for a more comprehensive understanding of the epidemiology of the HDV in order to plan therapeutic strategies.

Screening for the antibody to the HDV (anti-HD) has become generally available with several Enzyme Immuno Assays validated for specificity and sensitivity [12,13], but confirmation of active HDV infection by the detection of serum HDV RNA in RT-PCR is not generally available, and out of reach of the resources of many countries where the prevalence of HDV is high.

## 3. The Changing Epidemiological Scenario

The scenario of HDV has changed since the 1980s due to the worldwide implementation of HBV vaccination. In domestic populations of high-income countries, where vaccination and social progress are of long standing, HBV infection has much diminished, leading by default to a dramatic reduction of the circulation of the HDV [14]. Italy has been central to the unfolding of HDV changes, and the historical trend of the infection in the country is paradigmatic. The decrease of the infection has been age-dependent, with a consistent shift to the older age-groups. The mean age of anti-HD positive subjects increased from 38.1 years in 1987 to 51.7 years in 2019 and, at this time, only 2.6% of native-Italians were younger than 30 years [15]. The clinical pattern in the few thousands residual patients is an advanced fibrotic/cirrhotic liver disease, which maintains an impact on liver transplantation programs [16]. HDV has also diminished in drug-addicts [17,18], but these subjects still remain the major victims and source of the infection. In a recent metanalysis, the prevalence of anti-HD was more than three times higher in HBsAg drug users than in subjects without risk factors (37.6% vs. 10.6%) [9]. In Italy the proportion of acute hepatitis D in drug abusers diminished from 45.5% in 1990–1999 to 4.2% in 2010–2019 but addicts had a 8.7-fold risk (C.I.95% = 5.3–14.4) of acquiring the HDV after adjustment for confounders [19].

In the last decades, the perception that hepatitis D is vanishing has diminished alert to HDV. However, immigrants from HDV-endemic areas coming as a labour force are re-introducing the infection, posing a new and important public health challenge [20,21]. Among chronic HBsAg carriers resident in Italy, those born abroad in 2019 were six times more likely to have anti-HD and had a younger median age than Italians (46 years versus 57 years; *p* < 0.01) [15]. A similar pattern of decreasing domestic and increasing migrant HDV infections is emerging throughout all high-income countries [22,23,24,25]. In the US the number of HBsAg carriers susceptible to the HDV superinfection is low and hepatitis D is not perceived as a problem. Nevertheless, in HBsAg carriers the prevalence of anti-HD was recently as high as 42% [26]; most of them drug addicts or foreign-born.

HBV vaccination is gradually reducing hepatitis D in many other countries of the world, but the epidemiology of HDV is changing at a slower and more variable pace than in Europe, depending on national resources, the diverse background of HBV infections and the availability of infrastructures for the effective delivery of the vaccine. As a tangible return, severe and fulminant outbreaks of juvenile hepatitis D were no longer reported in hyperendemic HDV countries. Eight years after the introduction of HBV vaccination, hepatitis D has been eliminated in children less than 11 years of age in indigenous populations of the Peruvian Amazon [27].

While HDV infection is coming under control in the developed world, its medical impact remains high in many disadvantaged countries of Africa and Asia where the prevalence of HBsAg carriers often exceeds 5% [14].However, the local burdens of hepatitis D remain ill-defined. In some countries the role of hepatitis D is still unknown for lack of testing, and in many others the reported prevalences are often inconsistent. Mongolia, Uzbekistan and Pakistan have among the highest rates of hepatitis D in the world, yet in neighbouring China and India the role of HDV is apparently negligible [14]. Likewise, many hot spots have emerged in Africa, yet throughout the same and adjacent countries of the continent the rates of the infection are often conflicting, and prevalence figures are unreliable.

## 4. What Lies Ahead

The price extolled by globalisation in the reconstitution of an HDV reservoir to Europe through increasing migratory fluxes should be recognized, and awareness of this new threat should enforce more vigilance to avoid HDV returning to the continent. First degree relatives and sexual partners of immigrants with hepatitis D infection should be tested for HBV markers, and vaccinated if they are negative for these markers. The most important task is to raise awareness of the problem of hepatitis D in Africa and Asia. A trivial but relevant factor that conceals the true impact of HDV is lack of testing, and it is therefore urgent that international health agencies establish and expand national screening efforts and promote data collection to provide accurate contemporary scenarios of HDV. In order to obtain reliable figures and overcome discrepancies in the epidemiology of HDV, several confounding factors need to be considered. While there is a strong correlation between anti-HD and chronic HDV liver disease, the antibody is not only a marker of HDV damage but it can also be found in healthy HBsAg carriers as a serologic scar to past HDV infections. However the percentage of HBsAg carriers with anti-HD who have HDV-RNA in blood and liver disease is about ten-fold higher than that of anti-HD positive healthy carriers with past HDV infections [28]. This means that the interpretation of anti-HD must be put in perspective with the type of population recruited for the testing.

In Africa and Asia, screening for anti-HD is often performed for convenience in asymptomatic HBsAg-carriers at small risk of HDV, such as those recruited at blood banks, in working and social communities, in pregnancy consultations, etc. This results in low prevalence figures which would indicate that hepatitis D is only of minor local concern. However, when the antibody is determined at medical centers in carriers with advanced liver disease, the rate of HDV increases significantly, indicating that the overall medical burden of hepatitis D is best determined from surveys for anti-HD in HBsAg cirrhotics [28].

Further confounders are that information comes most often from a single, or few, studies of small sample size used to make generalized statements, and that unaccounted regional differences may exist in the geographical distribution of HDV. Therefore, country prevalence estimates remain uncertain, as slight variations in patient numbers and study logistics can significantly change national prevalence figures. In order to assess the burden of HDV, national surveys should not only be centered on patients with advanced liver disease but these should be recruited at different regional sites within a country, in particular in the largest countries.

Though there is no prospect of a vaccine directed against HDV to prevent superinfection in HBsAg carriers, ordinary HBV vaccination offers a simple, cheap and effective protection against HDV to HBV-naïves, and vaccination campaigns should be implemented with priority in regions and communities where HDV is highly endemic, even though it will take decades to globally eliminate hepatitis D.

The therapy of chronic hepatitis remains a difficult task, as the unique nature of HDV is a hurdle to the development of specific therapies [29]. The time-honored therapy with interferon is of limited efficacy, and new therapeutic options in development [30] are promising but do not appear, so far, to be able to eradicate the HDV. Whether their performance will be improved by long-term therapy or by combination with Pegylated-IFN awaits the results of ongoing Phase 3 trials. In view of the geographical and social prevalence of HDV infections in poor areas, the global success of therapies in containing HDV will ultimately depend on whether they become affordable at low cost and accessible to delivery to the less affluent countries of the world, which are most in need of them.
